# A Novel Prognostic Model Based on the Serum Iron Level for Patients With Early-Stage Triple-Negative Breast Cancer

**DOI:** 10.3389/fcell.2021.777215

**Published:** 2021-11-04

**Authors:** Xin Hua, Fangfang Duan, Jiajia Huang, Xiwen Bi, Wen Xia, Chenge Song, Li Wang, Chang Jiang, Zhongyu Yuan

**Affiliations:** Department of Medical Oncology, The State Key Laboratory of Oncology in South China, Collaborative Innovation Center for Cancer Medicine, Sun Yat-sen University Cancer Center, Guangzhou, China

**Keywords:** serum iron level, early-stage triple-negative breast cancer, predictive model, nomogram, survival

## Abstract

The dysregulation of iron homeostasis has been explored in malignancies. However, studies focusing on the association between the serum iron level and prognosis of patients with early-stage triple-negative breast cancer (TNBC) are scarce. Accordingly, in current study, 272 patients with early-stage TNBC treated at Sun Yat-sen University Cancer Center (SYSUCC) between September 2005 and October 2016 were included as a training cohort, another 86 patients from a previous randomized trial, SYSUCC-001, were analyzed as a validation cohort (SYSUCC-001 cohort). We retrospectively collected their clinicopathological data and tested the serum iron level using blood samples at the diagnosis. In the training cohort, patients were divided into low-iron and high-iron groups according to the serum iron level cut-off of 17.84 μmol/L determined by maximally selected rank statistics. After a median follow-up of 87.10 months, patients with a low iron had a significantly longer median disease-free survival (DFS) of 89.13 [interquartile range (IQR): 66.88–117.38] months and median overall survival (OS) of 92.85 (IQR: 68.83–117.38) months than those in the high-iron group (median DFS: 75.25, IQR: 39.76–105.70 months, *P* = 0.015; median OS: 77.17, IQR: 59.38–110.28 months, *P* = 0.015). Univariate and multivariate Cox analysis demonstrated the serum iron level to be an independent predictor for DFS and OS. Then, a prognostic nomogram incorporating the serum iron level, T stage and N stage was developed for individualized prognosis predictions. It had good discriminative ability with a C-index of DFS (0.729; 95% CI 0.666–0.792) and OS (0.739; 95% CI 0.666–0.812), respectively. Furtherly, we validated the predictive model in the SYSUCC-001 cohort, which also showed excellent predictive performance with a C-index of DFS (0.735; 95% CI 0.614–0.855) and OS (0.722; 95% CI 0.577–0.867), respectively. All these suggested that the serum iron level might be a potential prognostic biomarker for patients with early-stage TNBC, the predictive model based on it might be served as a practical tool for individualized survival predictions.

## Introduction

Triple-negative breast cancer (TNBC), lacking the expression of estrogen receptor (ER), progesterone receptor (PR), and human epidermal growth factor receptor 2 (HER-2), accounts for about 10–15% and is the most aggressive molecular subtype of all breast tumors (Sung et al., 2021; Wang et al., 2021). Due to its invasiveness, early relapse, strong heterogeneity and limited therapeutic options, TNBC patients usually have distant metastasis at the diagnosis and worse long-term clinical outcomes compared with patients with other subtypes of breast cancer (Gadi and Davidson, 2017; Vagia et al., 2020). In recent years, advances in the landscape of diagnosis and treatment have elicited survival benefits for patients with TNBC. Nevertheless, TNBC remains a huge threat to life due to its recurrence and relatively high mortality (Dent et al., 2007; Bianchini et al., 2016; Garrido-Castro et al., 2019). Thus, identification of novel, accurate biomarkers, and exploration of individualized therapeutic targets for women with TNBC is very necessary.

Iron is a critical trace element for the activity of many proteins and enzymes. Iron is involved in cell respiration, oxygen transport, energy metabolism, DNA repair, and different signaling pathways (Torti et al., 2018). It is essential for human health, but excess iron or iron overload due to disorders of iron metabolism can induce severe toxicity even tumorigenesis in humans (Wu et al., 2004; Adams, 2015).

Increasing studies have demonstrated an association between consumption of red meat, intake of heme iron, or dietary intake of iron and initiation of breast cancer (Ferrucci et al., 2009; Guo J. et al., 2015; Inoue-Choi et al., 2016). Also, dysregulation of systemic iron homeostasis is a risk factor for the initiation, growth, progression, and metastasis of tumor cells (Bingham et al., 2002; Radulescu et al., 2012; Guo et al., 2015). Besides, iron accumulation has an important role in multiple cell-death pathways, including iron-dependent cell death, i.e., ferroptosis, which suggests a potential therapeutic target to inhibit tumor development in cancer patients (Dixon et al., 2012; Basuli et al., 2017). Several preclinical/clinical studies have explored the anti-tumor activity and safety of depleting iron overload in tumors by means of iron chelators (Nutting et al., 2009; Yamasaki et al., 2011; List et al., 2012; Neufeld et al., 2012; Kalinowski et al., 2016). Targeting an increased iron level instead of iron chelators could also be a novel treatment option (Stockwell et al., 2017; von Hagens et al., 2017). Usually, researchers obtain values for iron by measuring circulating levels of iron-bound proteins (e.g., transferrin, ferritin) (Hambidge, 2003; Fonseca-Nunes et al., 2014; Morales and Xue, 2021). However, direct measurement of iron is more accurate to reflect the iron level in the body, but is poorly understood.

Thus, we explored the association between the baseline serum iron level at the diagnosis and clinical prognosis of women with early-stage TNBC. We aimed to establish a model on basis of direct measurement of the serum iron level for individualized prognosis predictions and treatment guidance.

## Materials and Methods

### Study Design and Patient Eligibility

We retrospectively analyzed the prognostic value of the serum iron level in women newly diagnosed with TNBC between September 2005 and October 2016 at Sun Yat-sen University Cancer Center (SYSUCC) in Guangzhou, China. Approval for our study protocol was obtained from the Ethics Committees of SYSUCC (2021-FXY-140). The requirement for written informed consent from patients were waived due to the retrospective nature of our study. We processed all personal data anonymously following the Helsinki Declaration 1964 and its later amendments.

The inclusion criteria were: (i) age ≥ 18 years; (ii) breast cancer diagnosed by pathology; (iii) hormone receptor-negative [<1% or 0 by immunohistochemical (IHC) staining in nuclei] according to American Society of Clinical Oncology (ASCO)/College of American Pathologists (CAP) guideline (Allison et al., 2020) and HER2-negative (scored as 0, 1 +, or 2 + by IHC analyses without amplification of the ERBB2 gene on fluorescence *in situ* hybridization) disease; (iv) patients were restaged at T1-4N0-3M0 according to the seventh version of the American Joint Committee on Cancer (AJCC 2010); (v) complete clinicopathological information and blood samples obtained within 1 week of the diagnosis.

Patients were excluded if they met the following criteria: (i) local relapse or distant metastasis at the diagnosis (i.e., brain, lung, bone, liver); (ii) pregnancy; (iii) previous malignancy including breast cancer; (iv) severe or uncontrolled complications.

### Data Collection and Measurement of Serum Iron Level

We retrieved the clinicopathological information of enrolled patients from the electronic medical records system of SYSUCC. We obtained their blood samples within 1 week of initiation of any anti-cancer treatment from the Tumor Resource Library of SYSUCC. Measurement of the serum iron level of participants was conducted using the Iron (Fe) Assay Kit (PAESA Chromogenic Method) of the Cobas 8000 system (Roche Diagnostics, Basel, Switzerland).

### Follow-Up and Endpoints

Follow-up data were obtained using the outpatient electronic records of SYSUCC or telephone interviews. Patients were evaluated every 3 months within 2 years of the diagnosis, then every 6 months until 5 years and, subsequently, annually. The assessment comprised routine hematology and laboratory tests, menopausal status, ultrasound (breast, abdomen) or computed tomography. Radiography and bone scintigraphy were undertaken annually.

The primary endpoint was disease-free survival (DFS), which was defined as the time from the diagnosis to the first disease progression or death due to any cause. The second endpoint was overall survival (OS), which was defined as the time from the diagnosis to death due to any cause.

### Statistical Analysis

Age is shown as the median with interquartile range (IQR). Categorical variables are listed as frequencies with percentages. The cutoff for the serum iron level was determined by maximally selected rank statistics using the “maxstat” plugin (R Institute of Statistical Computing, Vienna, Austria). We stratified patients with early-stage TNBC into low- and high-iron groups. Survival curves of these two groups were estimated by the Kaplan–Meier method and compared using the log-rank test. If *P* < 0.05 was achieved in the univariate Cox regression model, factors could be analyzed further in the multivariate Cox proportional hazards analysis. Factors were examined according to the Schoenfeld residuals (Wileyto et al., 2013), and their corresponding hazard ratios with 95% confidence interval (CIs) were estimated. Subsequently, a prognostic nomogram incorporating the serum iron level with other independent clinicopathological indicators was developed. The discrimination performance of the predictive nomogram was assessed by the Concordance Index (C- index), calibration curves, and time-dependent receiver operating characteristic (ROC) curves in the training cohort and validation (SYSUCC-001) cohort. *P* < 0.05 (two-sided) was considered significant. Statistical analyses were conducted using R 4.0.1.

## Results

### Patients Clinicopathologic Characteristics in the Training Cohort

After excluding 75 women due to incomplete data (30 without the Ki67 Index; 38 without the histology grade; four without lymphovascular invasion; three without the T stage). Finally, a total of 358 patients with early-stage TNBC were eligible: 272 in the training cohort and 86 in the SYSUCC-001 cohort. The clinicopathological characteristics of patients are listed in Table 1.

**TABLE 1:** Characteristics of eligible patients.

Characteristic	ALL	Training cohort	SYSUCC-001
	*N* = 358	*N* = 272	*N* = 86
**Age (years), median (IQR)**	46.8 (39.3–56.0)	48.0 (41.0–49.2)	43.5 (37.3–50.8)
Age at the diagnosis (years)			
< 50	208 (58.1%)	149 (54.8%)	59 (68.6%)
≥ 50	150 (41.9%)	123 (45.2%)	27 (31.4%)
**T stage ^a^**			
T1	121 (33.8%)	92 (33.8%)	29 (33.7%)
T2	204 (57.0%)	151 (55.5%)	53 (61.7%)
T3	25 (7.0%)	23 (8.5%)	2 (2.3%)
T4	8 (2.2%)	6 (2.2%)	2 (2.3%)
**N stage ^a^**			
N0	208 (58.1%)	156 (57.4%)	52 (60.5%)
N1	82 (22.9%)	63 (23.2%)	19 (22.1%)
N2	34 (9.5%)	28 (10.3%)	6 (6.9%)
N3	34 (9.5%)	25 (9.1%)	9 (10.5%)
**Menopausal status**			
Premenopausal	242 (67.6%)	174 (63.8%)	68 (79.1%)
Postmenopausal	116 (32.4%)	98 (36.2%)	18 (20.9%)
**Pathological grade ^b^**			
1	6 (1.7%)	5 (1.84%)	1 (1.1%)
2	130 (36.3%)	114 (41.9%)	16 (18.6%)
3	213 (59.5%)	153 (56.2%)	60 (69.8%)
4	9 (2.5%)	0 (0.0%)	9 (10.5%)
**Ki-67 Index ^c^**			
< 30%	75 (20.9%)	63 (23.2%)	12 (14.0%)
≥ 30%	283 (79.1%)	209 (76.8%)	74 (86.0%)
**Lymphovascular invasion**			
No	284 (79.4%)	213 (78.3%)	71 (82.6%)
Yes	74 (20.6%)	59 (21.7%)	15 (17.4%)
Serum iron (μmol/L) ^d^, median (IQR)	14.7 (10.9–18.8)	14.7 (11.1–18.9)	14.6 (10.6–18.9)
**Serum iron (μmol/L) ^d^**			
High	114 (31.8%)	88 (32.4%)	26 (30.2%)
Low	244 (68.2%)	184 (67.6%)	60 (69.8%)

*^*a*^Diagnosed based on the AJCC 2010 criteria (seventh edition).*

*^*b*^Histological grade at the diagnosis was based on the degree of tumor differentiation.*

*^*c*^The Ki-67 index at the diagnosis indicates DNA synthetic activity as measured using immunocytochemistry.*

*^*d*^The cut-off value was determined by means of maximally selected rank statistics. IQR, interquartile range.*

In the training cohort, the median age was 48.0 (IQR 41.0–49.2) years. A total of 174 (63.8%) women were premenopausal and 98 (36.2%) were postmenopausal. Most patients (76.8%) had Ki-67 Index ≥ 30%. Also, 92 (33.8%), 151 (55.5%), 23 (8.5%), and 6 (2.2%) patients had a pathological stage of T1, T2, T3, and T4, respectively. In addition, 156 (57.4%) had the N0 stage, whereas N1, N2, and N3 stages accounted for 63 (23.2%), 28 (10.3%), and 25 (9.1%), respectively.

### Optimal Cut-Off Value of Serum Iron Level in the Training Cohort

**FIGURE 1:**
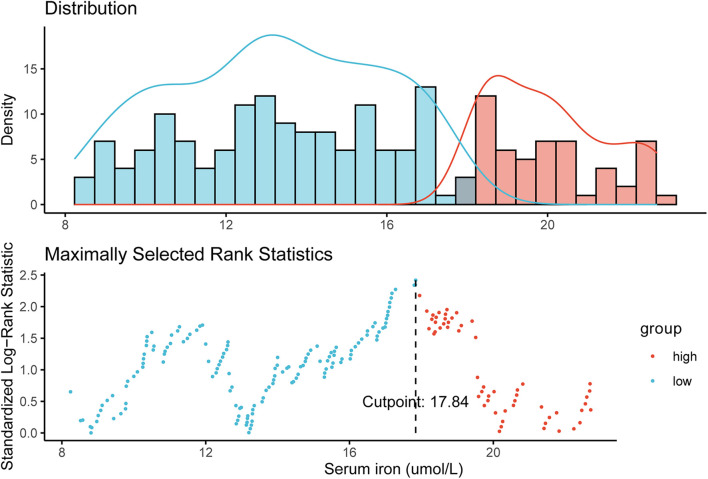
Definition of the cutoff of 17.84 μmol/L for the serum iron level according to maximally selected log-rank statistics.

We defined 17.84 μmol/L as the optimal cutoff of the serum iron level to stratify patients into two different iron groups according to maximally selected rank statistics (Figure 1). Eighty-eight (32.4%) women were classified into the high-iron group with serum iron > 17.84 μmol/L, and the other 184 (67.6%) patients had serum iron ≤ 17.84 μmol/L (Table 1).

### Survival Outcomes in the Training Cohort

**FIGURE 2:**
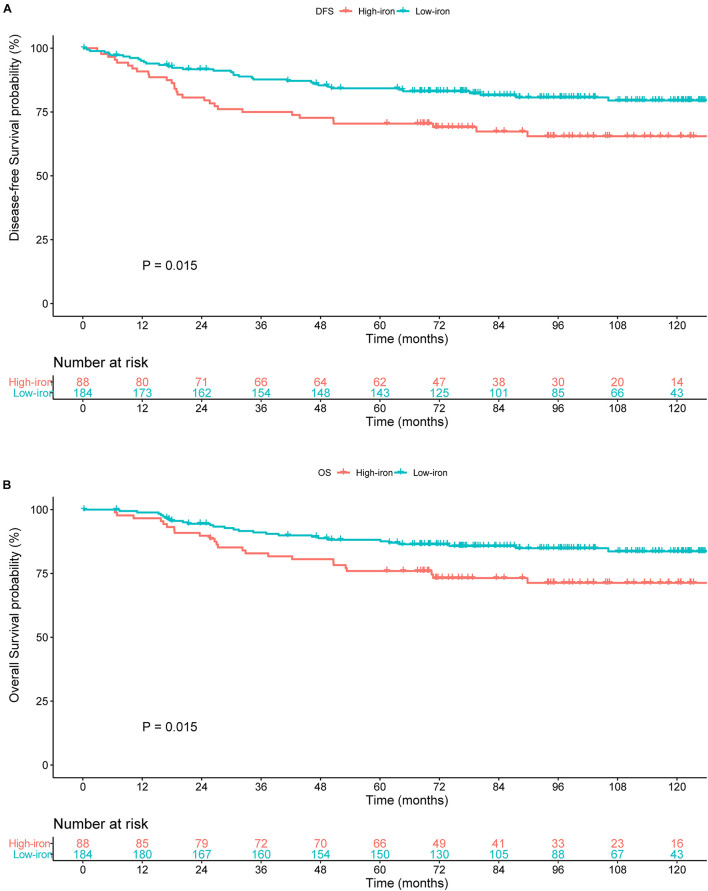
Survival curves estimated by the Kaplan–Meier method and compared with log-rank tests in different groups of serum iron level in the training cohort. **(A)** Disease-free survival (DFS) curve. **(B)** Overall survival (OS) curve. Low iron: ≤ 17.84 μmol/L; high iron: > 17.84 μmol/L.

The median duration of follow-up was 87.10 months. In the training cohort, compared with patients with a low serum iron level, patients with early-stage TNBC in the high-iron group achieved significantly shorter median DFS (89.13, IQR: 66.88–117.38 months vs. 75.25, IQR: 39.76–105.70 months, *P* = 0.015) (Figure 2A) and median OS (92.85, IQR: 68.83–117.38 months vs. 77.17, IQR: 59.38–110.28 months, *P* = 0.015) (Figure 2B), respectively.

### Development of the Prognostic Model

**FIGURE 3:**
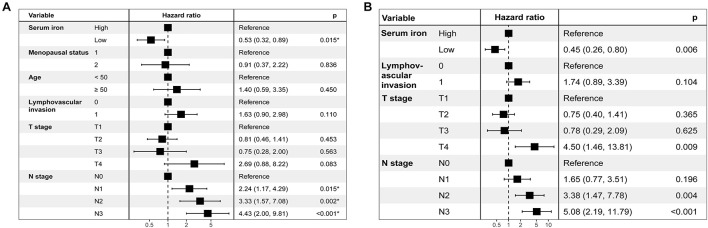
Results of stepwise multivariate Cox models in the training cohort are showed as forest plots. **(A)** Forest plot of disease-free survival (DFS). **(B)** Forest plot of overall survival (OS).

**FIGURE 4:**
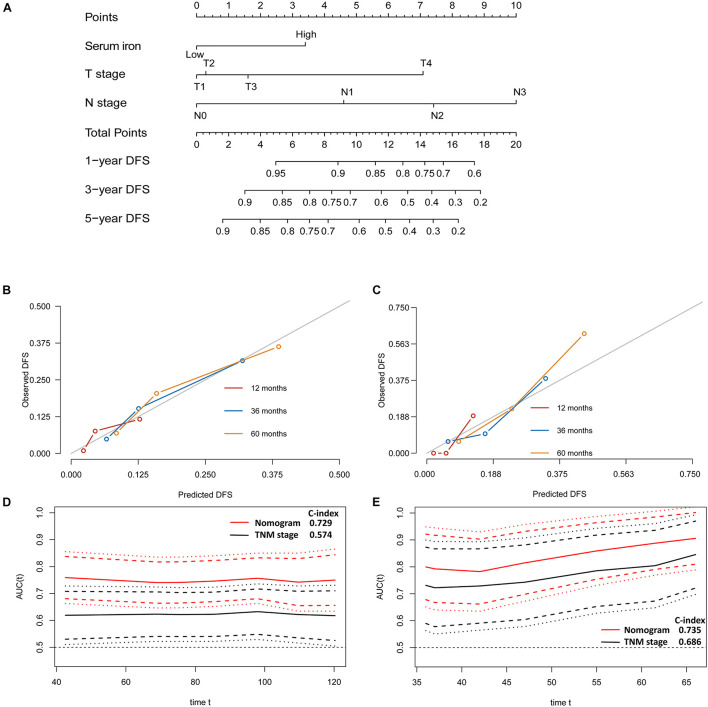
Establishment and validation of a model for individualized prediction of disease-free survival (DFS). **(A)** Nomogram of this predictive model for patients with early-stage triple-negative breast cancer. **(B)** Calibration plots of 1−, 3−, and 5-year DFS predictions in the training cohort. **(C)** Calibration plots of 1−, 3−, and 5-year DFS predictions in the SYSUCC-001 validation cohort. **(D)** Time-dependent receiver operating characteristic (ROC) curves in the training cohort. **(E)** Time-dependent ROC curves in the SYSUCC-001 cohort.

Table 2 shows results of the univariate Cox analysis for DFS in the training cohort. Variates achieved the predetermined significance (*P* < 0.05) in the univariate Cox regression model. Hence, age, menopausal status, lymphovascular invasion, T stage, N stage, and serum iron level were entered into the multivariate Cox analysis. The latter demonstrated that the N stage and serum iron level continued to be related significantly to DFS in patients with early-stage TNBC (Figure 3A). Then, a prognostic model incorporating the T stage, N stage, and serum iron level was established to predict DFS individually (Figure 4A).

**TABLE 2:** Univariate Cox regression analysis of overall survival and disease-free survival in women with breast cancer in the training cohort.

Characteristics	Overall survival		Disease-free survival	
	Hazard ratio (95%CI)	*P*	Hazard ratio (95%CI)	*P*
**Age (year)**				
< 50	Reference		Reference	
≥ 50	**2.039 (1.162–3.579)**	0.013*	**1.732 (1.060–2.830)**	0.029*
**Menopausal status**				
Premenopausal	Reference		Reference	
Postmenopausal	**1.826 (1.054–3.163)**	0.032*	**1.625 (0.998–2.647)**	**0.051**
**Histological grade ^a^**				
½	Reference		Reference	
3	1.246 (0.734–2.116)	0.415	1.098 (0.670–1.723)	0.685
**Lymphovascular invasion**				
No	Reference		Reference	
Yes	**2.752 (1.555–4.870)**	0.001*	**2.513 (1.491–4.236)**	0.001*
**Ki-67 index at diagnosis < 30% ^b^**				
No	Reference		Reference	
Yes	1.272 (0.637–2.539)	0.496	1.085 (0.606–1.943)	0.783
**T stage^c^**				
1	Reference		Reference	
2	0.854 (0.459–1.590)	0.619	0.826 (0.483–1.412)	0.484
3	1.412 (0.560–3.601)	0.419	1.062 (0.432–2.610)	0.896
4	**5.034 (1.691–14.990)**	0.004*	**3.496 (1.206–10.132)**	0.021*
**N stage ^c^**				
0	Reference		Reference	
1	1.882 (0.899–3.941)	0.094*	**2.416 (1.283–4.546)**	0.006*
2	**3.571 (1.634–7.804)**	0.001*	**3.582 (1.761–7.284)**	<0.001*
3	**6.669 (3.174–14.014)**	<0.001*	**6.767 (3.419–13.393)**	<0.001*
**Serum iron level (μmol/L) ^d^**				
High	Reference		Reference	
Low	**2.560 (1.363–4.811)**	0.017*	**0.550 (0.337–0.897)**	0.017*

*P<0.05.

*^*a*^Histological grade at the diagnosis was based on the degree of tumor differentiation.*

*^*b*^The Ki-67 index at the diagnosis indicates DNA synthetic activity as measured using immunocytochemistry.*

*^*c*^Diagnosed based on the AJCC 2010 criteria (seventh edition).*

*^*d*^The cut-off value was determined by maximally selected rank statistics.*

*The bold values represents that features reach to the predetermined significance threshold (*P* < 0.05) in the univariate Cox regression model.*

**FIGURE 5:**
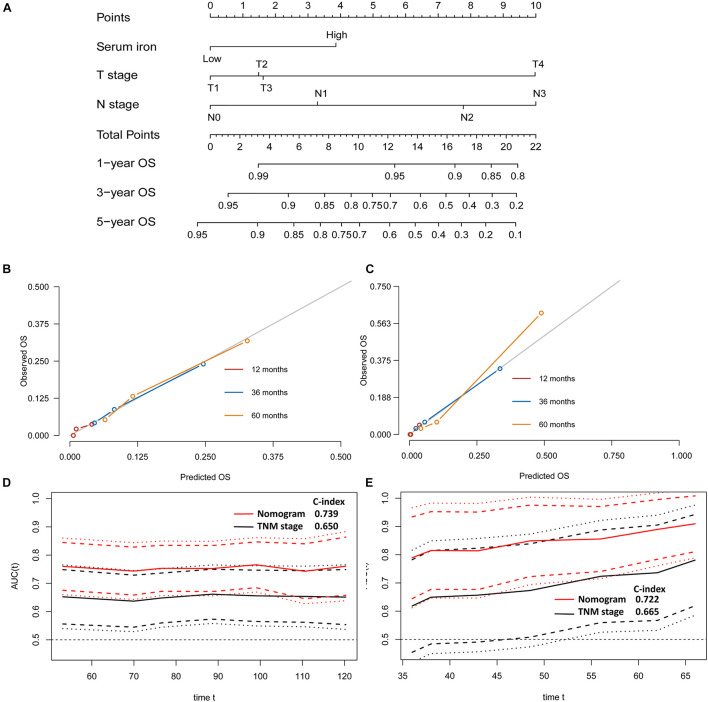
Establishment and validation of a model for individualized prediction of overall survival (OS). **(A)** Nomogram of this predictive model for patients with early-stage triple-negative breast cancer. **(B)** Calibration plots for predicting OS at 1−, 3−, and 5-year in the training cohort. **(C)** Calibration plots for predicting OS at 1−, 3−, and 5-year in the SYSUCC-001 validation cohort. **(D)** Time-dependent receiver operating characteristic (ROC) curves in the training cohort. **(E)** Time-dependent ROC curves in the SYSUCC-001 cohort.

Age, menopausal status, lymphovascular invasion, T stage, N stage, and serum iron level were recognized as independent predictors of OS for patients with early-stage TNBC (Table 2). Subsequently, the T stage, N stage, and serum iron level continued to be independent indicators for OS in patients with early-stage TNBC according to the multivariate Cox regression model (Figure 3B). On basis of the three independent prognostic factors stated above, we developed a model for individualized prediction of OS (Figure 5A).

### Evaluation of Predictive Performance of the Prognostic Model

The discriminative accuracy and prognostic ability of the prognostic nomogram of DFS were very good. It achieved a good C-index of 0.729 (95%CI 0.666–0.792) in the training cohort and 0.735 (95%CI 0.614–0.855) in the SYSUCC-001 cohort, respectively. Calibration plots for 1−, 3−, and 5-year DFS showed satisfactory consistency between the actual DFS and nomogram-predicted DFS in the training cohort and SYSUCC-001 cohort (Figures 4B,C). Time-dependent ROC curves suggested that the prognostic value of this nomogram for DFS was much better than that using the traditional tumor-node-metastasis (TNM) staging system in the training cohort (Figure 4D) and SYSUCC-001 cohort, respectively (Figure 4E).

The predictive nomogram of OS also had good discrimination with a satisfactory C Index of 0.739 (95%CI 0.666–0.812) in the training cohort and 0.722 (95%CI 0.577–0.867) in the SYSUCC-001 cohort, respectively. Good agreement between the observed 1−, 3−, and 5-year OS and nomogram-predicted 1−, 3−, and 5-year OS was documented in the calibration plot (Figures 5B,C). Moreover, compared with traditional TNM staging, the prognostic accuracy of this predictive nomogram in OS was more accurate based on the time-dependent ROC curves in the training cohort (Figure 5D) and SYSUCC-001 cohort, respectively (Figure 5E).

## Discussion

In this study, we determined a cut-off value of 17.84 μmol/L for the serum iron level to stratify heterogeneous female with early-stage TNBC into low- and high-iron groups according to maximally selected rank statistics. Patients in the high-iron group had a significantly shorter median survival than those in the low-iron group. Multivariate Cox regression analysis revealed that a high serum iron level continued to be an independent predicator of poor survival in patients with early-stage TNBC. Then, a prognostic model combining the serum iron level and two clinicopathological factors (T stage, N stage) was established and represented graphically as a nomogram. The latter showed satisfactory discriminative accuracy and good predictive consistency between the actual survival probability and nomogram-predicted clinical outcome in the training cohort and SYSUCC-001 cohort, respectively.

Iron is essential for the activity or inhibition of various proteins and enzymes involved in many biological processes (Adams, 2015; Torti et al., 2018). However, iron also contributes to oxidative stress, which can result in damage to DNA. Increasing numbers of studies have demonstrated that homeostatic dysregulation of iron metabolism and changes in distribution of iron in serum are found in different types of cancers, including breast cancer (Galaris and Pantopoulos, 2008; Torti et al., 2018). Excess iron or iron overload due to dysregulation of iron homeostasis can promote the development, progression, and metastasis of tumor cells (Bingham et al., 2002; Radulescu et al., 2012; Guo W. et al., 2015).

The main source of biologically available iron comes from dietary intake. More and more studies have explored a positive relationship between the intake of red meat, heme iron, and initiation of breast tumors (Ferrucci et al., 2009; Guo J. et al., 2015; Inoue-Choi et al., 2016). Also, iron accumulation might have a significant role in multiple pathways of programmed cell death, including apoptosis, necroptosis, ascorbate-mediated death, and ferroptosis (Dixon et al., 2012; Basuli et al., 2017; Torti et al., 2018). Therefore, iron chelators, because of depleting iron levels in the body, have been investigated as a potential therapeutic strategy with promising outcomes for cancer patients (Nutting et al., 2009; Yamasaki et al., 2011; List et al., 2012; Neufeld et al., 2012; Kalinowski et al., 2016).

Excess iron can lead to lipid peroxidation, DNA/protein damage, as well as the initiation and progression of tumors (Galaris and Pantopoulos, 2008; Radulescu et al., 2012; Guo W. et al., 2015; Torti et al., 2018). Conversely, the toxicity of iron accumulation can promote lethal damage to tumor cells by peroxidation of membrane lipids, subsequently, contribute to interruption of tumorigenesis and tumor development (Wang et al., 2018; Chang et al., 2019). Hence, iron overload or iron depletion might provide potential targets for anti-tumor treatment. Therefore, exploring the relationship between iron levels in the body and cancer is a rational approach.

Most studies have assessed iron levels in the body by measuring circulating levels of iron-bound proteins (e.g., transferrin, ferritin) (Hambidge, 2003; Fonseca-Nunes et al., 2014; Morales and Xue, 2021). However, this strategy might generate errors in reflecting the actual iron level, and few studies have measured the iron level directly (Torti et al., 2018). In current study, we creatively measured the serum iron level rather than levels of transferrin or ferritin to represent the iron level in the body, and explored the prognostic value of serum iron levels, on which researches remain not to reach a consensus up to date. Feng et al. evaluated trace element levels in serum for patients with different types of cancer, they failed to find a significant difference of serum iron between liver, kidney tumors and normal tissues (Yang et al., 2021). Others researchers demonstrated that, compared with normal cells, tumor cells were more dependent upon iron, and that they remodeled iron-metabolism pathways to acquire, store, and efflux iron during their development and replication (Wang et al., 2018). Patients with cancer suffering from anemia due to cachexia or therapeutic drugs tend to have a low serum iron level. This phenomenon has been explored in several tumor types, including breast cancer (Torti et al., 2018; Yang et al., 2021). In this study, we collected baseline blood samples < 1 week of the diagnosis, and defined an optimal cutoff of 17.84 μmol/L for the serum iron level. Based on the latter, we stratified patients with early-stage TNBC into two groups with significantly different survival outcomes.

Based on classification of the serum iron level, a prognostic model incorporating the serum iron level as well as the traditional T stage and N stage was developed. The common 21-gene recurrence score, 70-gene MammaPrint Assay, and the PAM50 prognostic model are limited to a specific subtype or lymph node-negative breast cancer or patients at high clinical risk from breast cancer with limited predictive accuracy of the C-index (Gnant et al., 2015; Wallden et al., 2015; Ibraheem et al., 2020; Poorvu et al., 2020). Our predictive nomogram was accurate, cost-efficient, convenient, and readily available in hospitals in developing countries. The TNM staging system is used commonly for risk stratification and therapeutic recommendations. However, TNM criteria are based on a limited number of clinical factors, and their discriminative accuracy is limited due to differences between patients (Bareche et al., 2018; Grosselin et al., 2019). According to our time-dependent ROC curves, the predictive accuracy of our prognostic model was higher than that of the traditional TNM staging system in the training cohort and SYSUCC-001 cohort, which suggests that our nomogram might be a potential supplement to the traditional TNM staging system. Besides, except from the TNM staging system, a series of prognostic models based on inflammatory status, tumor marker, stromal tumor-infiltrating lymphocytes, and kinds of gene signature have been explored with C-index ranging 0.69–0.77 (Shi et al., 2019; Yang et al., 2019; Zheng et al., 2020), compared with them, our prognostic models achieved a comparative predictive accuracy, and was more cost-efficient and convenient. As far as we know, our study is the first to propose a predictive model integrating the impact of trace element iron with clinicopathological features. But it should be noted that a further exploration about the preliminary mechanisms is warrant.

Our study had three main limitations. First, a retrospective study will have a selection bias. Nevertheless, we tried our best to enroll all eligible TNBC patients to minimize a selection bias, and validated our prognostic models in a cohort from previous randomized trial SYSUCC-001. Second, we measured only the baseline serum iron level at the diagnosis. It would have been preferable to monitor the dynamic change in the serum iron level during therapy and adjust the therapeutic strategy. Third, we included early-stage TNBC patients only from China. Hence, the availability and predictive accuracy of our prognostic nomogram to women from other geographic regions are required to be warrant in future study.

## Conclusion

We proposed a cutoff of the serum iron level to stratify patients with early-stage TNBC into high- and low-iron groups. On basis of the serum iron level, we established a predictive model for individualized survival prediction and validated it in the SYSUCC-001 cohort. The prognostic nomogram showed good predictive performance and satisfactory consistency compared with the actual clinical outcome.

## Data Availability Statement

The raw data supporting the conclusions of this article will be made available by the authors, without undue reservation.

## Ethics Statement

The studies involving human participants were reviewed and approved by the Ethics Committee of Sun Yat-sen University Cancer Center. The ethics committee waived the requirement of written informed consent for participation.

## Author Contributions

ZY designed this study. XH, FD, and JH collected, primarily analyzed, and interpreted data. XH, FD, JH, CS, LW, and CJ participated in the drafting of the manuscript. XH, FD, JH, WX, XB, and ZY contributed to administrative, technical, or material support. All authors revised this manuscript and approved the final submitted version.

## Conflict of Interest

The authors declare that the research was conducted in the absence of any commercial or financial relationships that could be construed as a potential conflict of interest.

## Publisher’s Note

All claims expressed in this article are solely those of the authors and do not necessarily represent those of their affiliated organizations, or those of the publisher, the editors and the reviewers. Any product that may be evaluated in this article, or claim that may be made by its manufacturer, is not guaranteed or endorsed by the publisher.
